# Knowledge, attitude, and practices (KAP) of patients with diabetes towards diabetes mellitus in Saudi Arabia

**DOI:** 10.25122/jml-2025-0047

**Published:** 2025-10

**Authors:** Rana Flimban, Wesam Abduljabbar, Ahmed Ragab

**Affiliations:** 1 Fakeeh College for Medical Sciences, Jeddah, Kingdom of Saudi Arabia; 2 Medical Laboratory Sciences Department, Fakeeh College for Medical Sciences, Jeddah, Kingdom of Saudi Arabia; 3 Mansoura College of Medicine, Mansoura, Egypt

**Keywords:** diabetes mellitus, KAP, socio-demographic factors, diabetes care

## Abstract

This study aimed to assess the knowledge, attitude, and practices (KAP) of patients with type 2 diabetes mellitus (T2DM) in Jeddah, Saudi Arabia. A cross-sectional study was conducted among 307 participants from four diabetes clinics in Jeddah. A validated Arabic questionnaire was used to assess participants’ KAP scores, which were then analyzed against demographic characteristics. The study participants had a mean age of 44 ± 6.9 years, with a higher proportion of women (52.8%) than men (47.2%). Almost half of the sample (45.9%) held university degrees, whereas only 1.6% were illiterate. The mean knowledge score was 10.03 ± 3.45, with women scoring marginally higher than males (10.15 ± 3.37 vs. 9.90 ± 3.54). Similarly, the mean attitude score was 56.16 ± 12.85, with women scoring higher (56.94 ± 12.97) compared to males (55.38 ± 12.73). However, male patients outperformed female patients in practice scores (11.28 ± 3.82 vs. 10.99 ± 3.69). Logistic regression analysis revealed significant associations between higher KAP scores and advanced education, longer duration since diabetes diagnosis, and having relatives with diabetes (*P* < 0.05). Additionally, single, widowed, and divorced participants had significantly lower attitude scores (*P* < 0.05). In addition, age and income levels also showed significant correlations with KAP scores (*P* < 0.05). Education level, age, disease duration, income, family history of diabetes, and marital status significantly influenced the KAP scores of patients with. These findings highlight the need for tailored diabetes education programs to enhance patients’ KAP and improve health outcomes.

## Introduction

Diabetes mellitus (DM) is a chronic metabolic disorder characterized by elevated blood glucose and disturbances in the metabolism of fats, proteins, and carbohydrates [1]. Its etiology involves genetic and environmental interactions, including central obesity and sedentary lifestyles, which lead to impaired insulin secretion or action [1-3]. Type 2 diabetes (T2DM) is the most prevalent form, followed by type 1, and is actually considered a major public health concern [2]. Globally, DM is the seventh leading cause of death [4], with Saudi Arabia ranking second in the Middle East and seventh worldwide in prevalence, affecting 23.1% of its population (over 5 million people) [5]. By 2026, diabetes is projected to affect 24.3% of Saudi adults [6].

The ever-increasing prevalence indicates gaps in public awareness [2,7], as adequate knowledge of diabetes correlates with better disease attitudes, management, reduced complications, and improved outcomes [1,4,8-11].

Recent studies in Saudi Arabia, however, reveal gaps in knowledge about diabetes management and complications, emphasizing the need for targeted proper education. A 2022 Jeddah study found only 38% of patients had adequate diabetes knowledge, with significant gaps in complication awareness [12]. In another study held in Riyadh, however, 52% of patients scored poorly on dietary knowledge [13], and a study in Makkah revealed that 44% of patients with diabetes underestimated the importance of lifestyle modification in diabetes management [14]. Some recently published reports documented that only 29% believed regular glucose monitoring was essential for disease control [5]. Moreover, some research on the Saudi population stated that less than 50% adhere properly to medication regimens [15]. The Saudi Health Interview Survey from 2023 [16] showed poor knowledge and attitude for diabetes management, as 67% of the interviewed population reported insufficient physical activity.

This study aimed to assess the knowledge and self-management practices of patients with T2DM in Jeddah, Saudi Arabia, and explore how demographic factors (e.g., age, gender, and education) influence these outcomes.

## Material and Methods

A cross-sectional study was conducted to assess the knowledge, attitude, and practice of Saudi patients with diabetes who visited the diabetes clinics at four different general hospitals in Jeddah, western region, Saudi Arabia, from August 2024 through December 2024.

A minimum required sample size of 327 was calculated based on the prevalence of diabetes in Saudi Arabia, using a 95% confidence level, a 5% margin of error, and an assumed population proportion of 23.1% [5]. The calculation was performed using a web-based sample size calculator [17]. Of the estimated sample, 307 participants completed the study. Informed consent was obtained from all participants prior to data collection. The survey began with a declaration of authenticity and a clear explanation of the study objectives and procedures. Participants were assured of their right to withdraw from the study at any time without consequence.

Inclusion criteria comprised Saudi patients diagnosed with T2DM, aged 18 years and above, of both sexes, and without any concurrent or recent acute complications of the disease.

Exclusion criteria included individuals younger than 18 years, patients with other types of diabetes, pregnant women, or those with existing diabetic complications.

Data were collected using a previously validated questionnaire adapted from a similar study [18]. Content validity was established by an expert panel comprising specialists in endocrinology, diabetology, and internal medicine to ensure the relevance and appropriateness of the items. Face validity was subsequently assessed through a pilot study involving ten participants, who were interviewed to evaluate clarity and comprehensibility. After receiving feedback and making all the required clarifications, the questionnaire was distributed on social networks in Arabic.

The questionnaire consisted of 35 items that measured knowledge, attitude, and practice of patients with TD2M. The questionnaire was divided into four sections: demographics, knowledge, attitude, and practice sections. Data collectors conducted brief web-based interviews before sharing the questionnaire through digital platforms to minimize researcher influence and ensure participant privacy.

The first part of the questionnaire focused on participants’ demographic characteristics, including age, gender, level of education, occupation, monthly income, time of diagnosis, duration of the disease, and medications included for treatment. Additionally, patients were asked whether their occupations and education were related to the medical field. In the second section of the survey, questions about risk factors, diagnosis, prevention, follow up and complications of diabetes mellitus were used to scale respondents' knowledge of the disease e.g. awareness about the types of diabetes, normal blood glucose level, follow up of glucose level and glycosylated hemoglobin, frequency of hypo or hyperglycemic attacks, frequency of diabetic comas, type and dose of medications used and neurological symptoms or vascular complications.

The knowledge section consisted of items with three response options: 'Yes.', 'No.', and 'I do not know.' Correct answers were assigned one point, while incorrect and 'I do not know' responses were given zero points, resulting in a total score ranging from 0 to 20. Knowledge levels were classified as low (0–10), moderate (11–15), or high (16–20). Another category of questions assessed respondents’ agreement with various behaviors related to diabetes in order to evaluate their perceptions and beliefs regarding diabetes management and lifestyle changes, e.g., whether treatment is used consistently, the amount and type of healthy food, whether this food affects the diabetic state or blood glucose level, how to test blood glucose levels, when and how to follow up the diabetic state, and the occurrence of systemic vascular or neurological complications. Each question was answered on a scale of 1–5, where 1 meant *strongly disagree* and 5 meant *strongly agree*. Based on the total number of attitude questions, scores ranged from 22 to 110. Scores below 50% of the total were considered low, 50–75% were considered moderate, and above 75% were considered high. Finally, the fourth part assessed practices in diabetes management by using specific questions related to consultations with health professionals about diabetes-related issues, diabetes screening, and participants’ practices in preventing or managing their diabetic state. Practice scores ranged from 0–16, where 0–8 was considered a low score, 8–12 was considered moderate, and above 12 was considered high.

For participants with limited literacy, the consent form was read aloud either to the participant or to their legally authorized representative. In all cases, the investigators provided sufficient time and opportunity for them to decide whether to participate. Responses to the questionnaire were based on information previously explained by the investigators, who also followed up during completion to assist and clarify any questions.

Data collected through online questionnaires was stored and managed securely on a password-protected server. Personal identifiers were removed from the dataset to protect participants' privacy. Data cleaning and validation procedures were conducted to identify and correct any errors or inconsistencies. The cleaned data were then exported from the web survey platform and imported into a secure electronic database for analysis using SPSS software version 26. This process ensured both data accuracy and security. The dependent variables were patients' knowledge, attitude, and practice, while the independent variables were socio-demographic characteristics.

### Statistical analysis

The Statistical Package for the Social Sciences (SPSS, version 26.0) was used to perform descriptive statistics (means, frequencies, and percentages) for knowledge, attitude, and practice (KAP) scores. Inferential statistics, including *t*-tests or ANOVA, were applied to compare KAP scores across demographic groups or other variables of interest where appropriate. Independent variables consisted of participants’ sociodemographic characteristics (e.g., age, gender, education level, income, marital status, and having a relative with diabetes), while the dependent variables were the KAP scores. Correlation analysis was conducted to assess relationships between KAP scores and continuous variables (e.g., age, education level, duration of diabetes), with 95% confidence intervals reported. Statistical significance was set at *P* < 0.05.

## Results

### Participant demographics

A total of 307 participants completed the questionnaire and were included in the final analysis. As shown in Table 1, the mean age (±SD) was 44.0 ± 6.9 years, with the largest age group being 41–50 years (22.1%) and the smallest 51–60 years (7.5%). The sample included slightly more females (52.8%) than males (47.2%). Marital status distribution was as follows: married (45.9%), divorced (30.6%), single (12.1%), and widowed (11.4%). Education levels varied, with the majority holding a university degree (45.9%), followed by postgraduate qualifications (23.1%). Illiteracy and primary education were the least reported (1.6% and 3.6%, respectively). Employment status was nearly evenly distributed: 22.5% were government employees, 22.5% self-employed, 21.8% private sector employees, 13.0% retired, and 20.2% unemployed. Monthly income distribution was as follows: <5,000 SAR (28.0%), 5,000–10,000 SAR (30.3%), 10,000–20,000 SAR (26.1%), and >20,000 SAR (15.6%). Regarding family history of diabetes, 57.7% were uncertain, 30.3% reported no history, and 12.0% confirmed a positive history.

**Table 1:** Socio-demographic characteristics of the participants (*n* = 307). Data presented in numbers (%)

Demographic variable	Count [*n*]	(%)
**Age group**		
18- 20 years	49	16.0%
20-30 years	55	17.9%
31-40 years	49	16.0%
41-50 years	68	22.1%
51-60 years	23	7.5%
More than 60 years	63	20.5%
**Gender**		
Females	162	52.8%
Males	145	47.2%
**Marital status**		
Single	37	12.1%
Married	141	45.9%
Divorced	94	30.6%
Widowed	35	11.4%
**Education level**		
Illiterate	5	1.6%
Primary School	11	3.6 %
Middle School	28	9.1 %
High School	51	16.6
University	141	45.9%
Postgraduate Studies	71	23.1
**Employment status**		
Government Employee	69	22.5%
Private Sector Employee	67	21.8%
Self-employed	69	22.5%
Retired	40	13.0%
Unemployed	61	20.2%
**Monthly income (Saudi Riyal)**		
Less than 5000	86	28.0%
5000-10000	93	30.3%
10000-20000	80	26.1%
More than 20000	48	15.6%
**Household size**		
Living alone	16	5.2%
1	70	22.8%
2	65	21.2%
3	44	14.3%
4	84	27.4%
5 or more	28	9.1%
**Relatives with diabetes**		
Yes	37	12 %
No	93	30.3%
I Don't Know	177	57.7%

### Descriptive KAP scores

As presented in Table 2, the mean overall knowledge score was 10.03 ± 3.45, with women scoring slightly higher (10.15 ± 3.37) than men (9.90 ± 3.54). For attitude, the mean score was 56.16 ± 12.85, with women again scoring higher (56.94 ± 12.97 vs. 55.38 ± 12.73). The mean practice estimated was 11.14 ± 3.75, with men scoring marginally higher (11.28 ± 3.82 vs. 10.99 ± 3.69).

**Table 2:** Association between mean ± SD of knowledge, attitude, and practice scores by sociodemographic characteristics

Demographic Variable	Level	Knowledge (Mean ± SD)	Knowledge *P* value	Attitude (Mean ± SD)	Attitude *P* value	Practice (Mean ± SD)	Practice *P* value
**Gender**	Female	10.15 ± 3.37	0.524	56.94 ± 12.97	0.566	10.99 ± 3.69	0.499
	Male	9.90 ± 3.54		55.38 ± 12.73		11.28 ± 3.82	
**Marital status**	Single	9.70 ± 3.56	0.133	56.13 ± 13.83	0.033	11.54 ± 4.00	0.882
	Married	10.47 ± 3.65		56.80 ± 12.63		11.00 ± 3.66	
	Divorced	9.22 ± 2.58		56.22 ± 13.11		11.14 ± 3.95	
	Widowed	10.00 ± 2.91		56.30 ± 11.23		11.29 ± 3.49	
**Education level**	Illiterate	8.35 ± 1.14	<0.001	48.40 ± 13.52	0.276	9.27 ± 4.15	0.337
	Elementary School	8.55 ± 2.54		51.18 ± 15.28		11.24 ± 4.05	
	Middle School	8.96 ± 2.40		53.07 ± 13.58		9.80 ± 4.38	
	High School	9.60 ± 3.24		54.61 ± 13.16		11.21 ± 3.51	
	Bachelor's	10.17 ± 3.48		57.81 ± 11.93	0.033	10.29 ± 4.52	
	Master’s/Doctorate	11.52 ± 3.49		57.44 ± 13.10		11.43 ± 3.55	
**Employment status**	Government Employee	10.04 ± 3.82	0.343	56.28 ± 13.25	0.46	10.80 ± 3.94	0.412
	Private Sector Employee	9.85 ± 3.15		55.63 ± 12.92		11.21 ± 3.82	
	Self-employed	9.46 ± 3.48		51.38 ± 14.12		11.25 ± 3.07	
	Retired	10.29 ± 3.50		56.75 ± 11.04		10.45 ± 4.13	
	Unemployed	10.35 ± 3.00		57.93 ± 13.08		11.70 ± 3.94	
**Monthly income (SAR)**	Less than 5000	9.34 ± 3.15	0.021	55.30 ± 12.45	0.261	10.71 ± 3.41	0.193
	5000-10000	9.38± 3.28		58.16 ± 12.23		11.08 ± 4.03	
	10000-20000	10.34 ± 3.64		55.98 ± 12.71		11.89 ± 3.73	
	More than 20000	11.15 ± 3.64		55.08 ± 14.84		10.79 ± 3.80	
**Relatives with T2DM**	Yes	10.32 ± 3.60	0.028	57.18 ± 13.20	0.181	10.32 ± 4.47	0.364
	No	9.78 ± 3.41		55.57 ± 12.38		11.18 ± 3.70	
	I Don't Know	9.27 ± 2.60		53.14 ± 12.12		11.29 ± 3.63	

Values are presented as mean ± SD. *P* values were calculated using *t*-tests for two-group comparisons and one-way ANOVA for variables with more than two groups, with post-hoc Tukey tests applied when applicable. Knowledge scores were based on 20 true/false items (range: 0–20), attitude scores on 22 Likert-scale items (range: 22–110), and practice scores were calculated from four Likert-scale items (range: 0–16). The sociodemographic data were tested for mean KAP scores, and P values were considered statistically significant when estimated below 0.05

### Bivariate associations and regression analysis

Key associations between KAP scores and sociodemographic factors were identified in Tables 2 and 3. As shown in Table 2, education level significantly influenced KAP scores, particularly for knowledge and attitude. Postgraduate participants recorded the highest knowledge and attitude scores (11.52 ± 3.49 and 57.44 ± 13.10, respectively), while those with no limited literacy scored the lowest (8.35 ± 1.14 and 48.40 ± 13.52, respectively; *P* < 0.001). In contrast, although postgraduates also had the highest mean practice score (11.43 ± 3.55), this difference was not statistically significant. Participants with a bachelor’s degree achieved the highest attitude score (57.81 ± 11.93; *P* = 0.033). In addition, participants with relatives diagnosed with T2DM demonstrated higher knowledge scores (57.18 ± 13.20; *P* = 0.028) compared with those without such family history. Income level was also found to affect knowledge scores, as participants earning <5,000 SAR had significantly lower scores (9.34 ± 3.15; *P* = 0.021) compared to higher-income groups. Employment status did not show any significant effect on KAP scores. However, marital status appeared to influence attitudes toward diabetes management, as single, divorced, and widowed participants reported lower attitude scores compared with married individuals (56.13 ± 13.83, 56.22 ± 13.11, and 56.30 ± 11.23; *P* = 0.033). Logistic regression analysis, as presented in Table 3, confirmed similar patterns. Higher knowledge scores were predicted by higher education level (95% CI; *P* < 0.001) and having a family history of diabetes (95% CI; *P* = 0.028). Increased practice scores were associated with older age (95% CI *; P* = 0.042), higher income level (95% CI *; P* = 0.004), and longer disease duration (95% CI; *P* < 0.001). Meanwhile, lower attitude scores were more likely among divorced or widowed participants (95% CI; *P* = 0.033).

**Table 3:** Association between KAP scores and sociodemographic characteristics by logistic regression analysis

Variable	Levels	Knowledge (B [95% CI]; *P*)	Attitude (B [95% CI]; *P*)	Practice (B [95% CI]; *P*)
**Sex (Reference: Female)**	Male	0.55 (–0.17, 1.27); 0.133	1.20 (–4.66, 2.26); 0.491	0.53 (–1.12, 2.17); 0.530
**Age (Years)**	—	0.003 (–0.03, 0.02); 0.818	0.10 (–0.04, 0.25); 0.174	0.08 (–0.15, –0.01); 0.042
**Marital status (Reference: Married)**	Single	0.04 (–0.96, 0.88); 0.933	0.07 (–3.76, 3.91); 0.979	1.82 (–4.25, 0.61); 0.136
	Divorced/Widowed	1.30 (–4.25, 0.64); 0.106	8.17 (–15.70, –0.64); 0.033	0.47 (–3.17, 4.10); 0.803
**Education (Reference: Has Degree)**	No Degree	1.66 (–2.37, –0.95); <0.001	3.04 (–7.06, 0.97); 0.135	0.33 (–1.63, 2.29); 0.736
**Employment (Reference: Employed)**	Unemployed	0.41 (–1.28, 0.46); 0.343	1.51 (–5.38, 2.36); 0.460	0.65 (–1.14, 2.45); 0.518
**Income (Reference: 0–4,999)**	5,000–9,999	0.31 (–1.23, 0.61); 0.502	1.93 (–2.38, 6.23); 0.382	0.45 (–1.63, 2.54); 0.004
	10,000–14,999	0.59 (–1.62, 0.45); 0.270	3.03 (–2.01, 8.07); 0.234	1.60 (–4.23, 1.02); .672
	15,000–19,999	0.87 (–2.01, 0.26); 0.131	0.09 (–5.20, 5.38); 0.974	3.85 (–6.43, –1.27); 0.197
	>20,000	0.67 (–0.93, 2.28); 0.413	2.43 (–10.01, 5.14); 0.531	2.05 (–5.76, 1.66); 0.279
**Years with T2DM**	—	0.10 (0.05, 0.15); <0.001	0.21 (–0.45, 0.03); 0.093	0.26 (0.13, 0.39); <0.001
**Relatives with T2DM (Reference: No)**	Yes	0.78 (0.08, 1.47); 0.028	2.24 (–1.01, 5.50); 0.181	1.20 (–0.74, 3.15); 0.222

Linear regression analysis was used to examine associations between socio-demographic factors and KAP scores. Values are unstandardized regression coefficients (B) with 95% confidence intervals in parentheses. Statistically significant predictors are estimated when *P* < 0.05.

## Discussion

The key findings of this study indicate that KAP scores varied significantly across the population based on sociodemographic characteristics. These results contribute to a deeper understanding of diabetes management and highlight the ongoing need for quality improvement initiatives and preventive strategies to reduce both short- and long-term complications of T2DM.

The mean knowledge score in this study was relatively low, indicating a significant concern among participants, with men exhibiting slightly lower scores than women. This finding is concerning, particularly given that previous national studies have reported higher levels of diabetes-related knowledge among patients with T2DM regarding the disease and its complications [19-22]. However, our results align with a 2018 systematic review by Alanazi *et al*. [23], which identified notable gaps in diabetes knowledge within the Saudi population, including healthcare professionals, medical students, and patients with diabetes. Additionally, similar findings have been reported in studies conducted in several Asian countries (China, Nepal, Bangladesh) and African countries (Benin) [24-27]. Although these findings may be expected in developing nations or rural populations [26,27], the persistence of low knowledge in this study suggests systemic educational shortcomings, warranting further investigation.

Postgraduate education strongly predicted higher knowledge scores, reinforcing prior national studies [3,21,22,28].

Disease duration and having a relative with diabetes were also associated with better awareness and adherence, and these findings are consistent with local [29,30] and international [31,32] research.

Participants' attitudes toward diabetes management were generally positive but varied according to sociodemographic factors. Married participants exhibited significantly higher attitude scores compared to single, divorced, or widowed individuals, suggesting the influence of social support and family structures on disease management perspectives. This observation is consistent with some research emphasizing the role of family and social networks in diabetes self-management [7,33-36]. In addition, positive attitudes toward diabetes among the study participants appeared to be influenced by both age and education level, corroborating earlier findings reported in international [18,37] and national studies [19,38].

Regarding diabetes-related practices, the mean practice score was moderate, yet a discrepancy between knowledge and practice was evident, particularly among low-income participants, though not among elderly individuals or those with long-standing diabetes. Such variability is consistent with global and regional findings [22,25,33,34,37,39,40], where high levels of knowledge do not consistently translate into improved diabetes management practices, particularly in areas such as dietary adherence and complications prevention. Similarly, Al-Wagdi and Al-Hanawi [7] found no direct correlation between good knowledge and behavior toward diabetes management. These findings strongly suggest the presence of psychosocial or systemic barriers (e.g., affordability, healthcare access) that may underlie this gap.

Finally, the results of the present study confirm that sociodemographic factors—including age, education, income, disease duration, and marital status—are significantly associated with KAP levels. These findings highlight the need for tailored interventions, such as addressing financial barriers to adherence, simplifying educational materials, and leveraging community health workers for outreach. Additionally, incorporating peer support groups can help mitigate the lack of familial support. To further bridge knowledge-practice gaps, healthcare provider training should emphasize patient-centered communication.

The authors acknowledge several limitations, including the cross-sectional design, which restricts the ability to establish causality, and the single-city sampling, which may limit generalizability. Future research should incorporate longitudinal and multi-regional studies to track KAP trends over time, while qualitative approaches are recommended to explore psychological and social barriers that hinder effective self-care.

## Conclusion

Sociodemographic factors, namely age, education, income, and marital status, significantly influence KAP among Saudi patients with T2DM. To enhance outcomes, policymakers should integrate diabetes education into primary care and community programs. Practice-level interventions should integrate knowledge building with practical support, while further research should explore structural barriers to bridging the knowledge-practice gap.
